# First person – Priyanka Mungara

**DOI:** 10.1242/dmm.052456

**Published:** 2025-05-23

## Abstract

First Person is a series of interviews with the first authors of a selection of papers published in Disease Models & Mechanisms, helping researchers promote themselves alongside their papers. Priyanka Mungara is first author on ‘ Urinary sodium wasting and disrupted collecting duct function in mice with distal renal tubular acidosis mutations’, published in DMM. Priyanka conducted the research described in this article while an MSc physiology graduate student in Dr Emmanuelle Cordat's lab at University of Alberta, Edmonton, Canada, investigating acid-base and sodium balance in mice with distal renal tubular acidosis (dRTA). She is now an MD student at University of Calgary, Calgary, Canada.


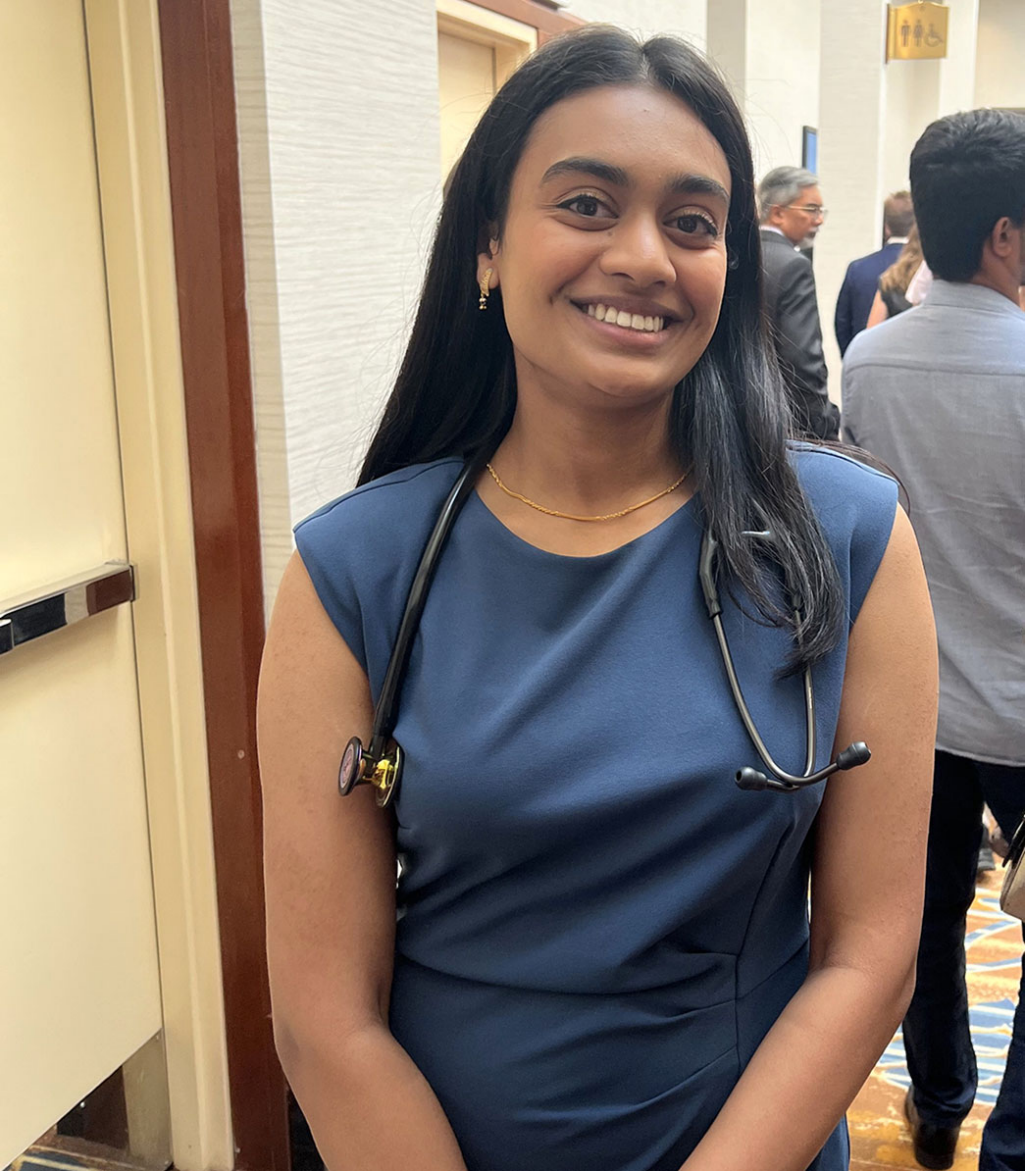


**Priyanka Mungara**

**Who or what inspired you to become a scientist?**

I was drawn to science because of its puzzle-like nature and the sense of exploration and curiosity it requires, qualities that align with my personality and interests! My inspiration to become a scientist stem from both my family and my PI, Dr Emmanuelle Cordat. My family instilled in me core values like hard work, curiosity and persistence, all of which are essential in scientific research. Dr Cordat's mentorship, support and expectations in the lab encouraged me to think critically, ask questions and investigate the answers.

**What is the main question or challenge in disease biology you are addressing in this paper? How did you go about investigating your question or challenge?**

The main challenge we aimed to address in this study was understanding why some patients with dRTA continue to exhibit a urinary sodium-wasting phenotype despite appropriate treatment. Given sodium's critical role in maintaining physiological balance, uncovering the mechanisms underlying this phenomenon could help inform more effective therapeutic strategies. To investigate this, we utilized kAE1 R607H and L919X mutant mouse models that mimic the human dRTA phenotype. We provided these mice a dietary challenge (salt-depleted acid load), which successfully reproduced the sodium-wasting phenotype observed in patients. To further explore the underlying mechanisms, we performed PCR and western blot analyses to assess the expression of key ion transporters across various nephron segments, aiming to understand how altered expression or regulation may contribute to this phenotype.

**How would you explain the main findings of your paper to non-scientific family and friends?**

In the nephron – part of the kidney that filters blood and balances salts and acid-base – ions are moved in two main ways: through cells (transcellular) or between the cells (paracellular). Normally, these two pathways work together and can compensate for each other when needed. In our study, we worked with mice that carry a genetic mutation to a protein that regulates acid-base balance in the nephron. We found that, due to this genetic mutation, normal ion movement through the cells was disrupted, thereby causing an increase in ion movement between the cells to compensate. In addition, we observed that an earlier part of the nephron, called the thick ascending limb, may also compensate to help maintain balance of these ions. Together, these findings highlight how the nephron is a highly complex and interconnected system. Even when one part is affected, other parts can adjust to help preserve the body's delicate internal balance.

[…] the study highlights a greater role for paracellular ion transport and the potential compensatory involvement of prior nephron segments to maintain acid-base and sodium balance.

**What are the potential implications of these results for disease biology and the possible impact on patients?**

These findings provide new insight into the mechanisms underlying the sodium-wasting phenotype observed in a subset of patients with dRTA. Specifically, the study highlights a greater role for paracellular ion transport and the potential compensatory involvement of prior nephron segments to maintain acid-base and sodium balance. This information deepens our understanding of the dRTA disease biology, emphasizing the adaptability of nephron function. Clinically, this knowledge may open the door to novel therapeutic strategies aimed at enhancing compensatory pathways, such as paracellular sodium and ion reabsorption in upstream nephron segments. Even in the absence of direct treatments, a more comprehensive understanding of renal ion handling can equip healthcare providers to make more informed decisions to support electrolyte balance and improve patient outcomes.

**Figure:**
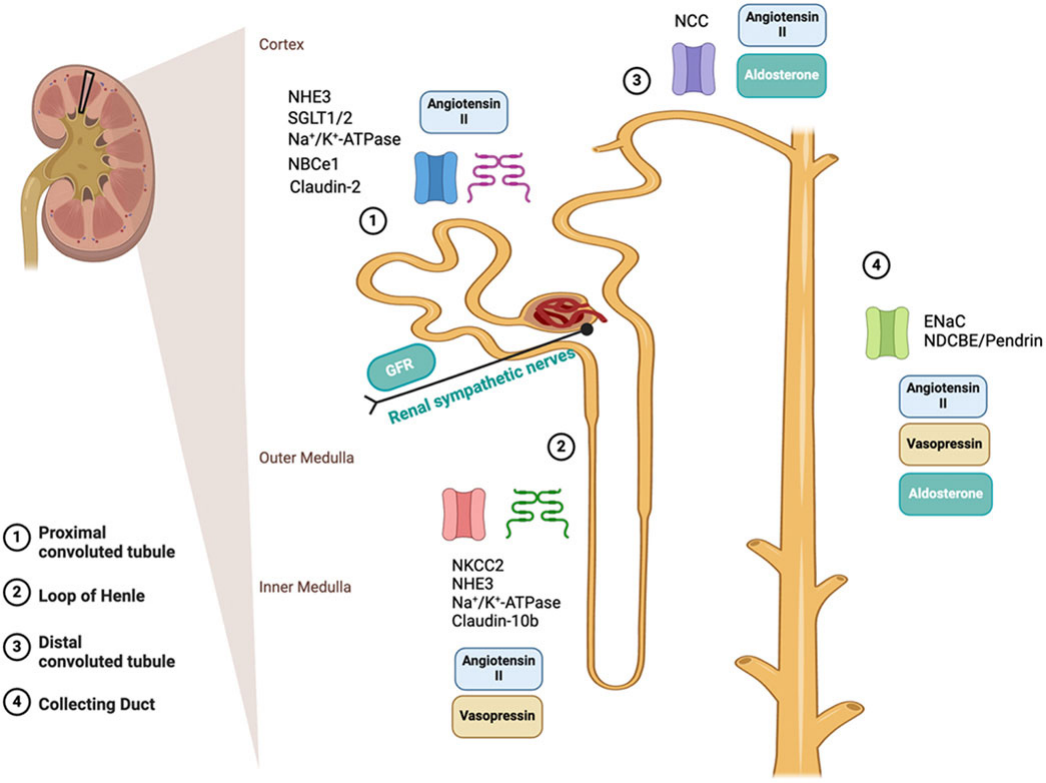
A visual guide on the transporters and influences of sodium transport along the nephron.

**Why did you choose DMM for your paper?**

DMM focuses on the use of model organisms to study human disease, which aligns very well with the aim of our research study. We used dRTA mutant mouse models that mimic the human dRTA phenotype to investigate disease mechanisms. As well, this journal is peer reviewed and fully open access, which increases the credibility and visibility of this work, allowing anyone interested to learn more about the field.

**Given your current role, what challenges do you face and what changes could improve the professional lives of other scientists in this role?**

As an MSc student, one of my biggest challenges was navigating the steep learning curve of becoming a researcher – balancing lab work, reading papers/studying and developing confidence in myself. To improve the experience of future scientists in this role, mentorship and peer support are essential. I was fortunate to have an amazing support system in my lab to help with troubleshooting, discussing progress and overall collaboration. Creating this type of supportive culture in labs would help the professional lives of other scientists in this role.

In terms of the research itself, a major challenge in kAE1 studies is the absence of physiologically relevant models to study kidney intercalated cells. We worked with two mutant mouse models that mimic the dRTA phenotype, but also show some difference to dRTA in humans. Creating viable kidney intercalated cell models and further kAE1 mutant *in vivo* models can better characterize mechanisms for dRTA pathology.

**What's next for you?**

I am currently a medical student at the University of Calgary. I am excited to continue exploring and contributing to research while at medical school. I hope to build on the skills and knowledge I developed in the lab and apply them in the meaningful ways – advancing research and caring for my future patients!

**Tell us something interesting about yourself that wouldn't be on your CV**

I grew up learning Indian classical music (Carnatic music) and dance (Bharatnatyam)!
